# Distorted correlations among censored data: causes, effects, and correction

**DOI:** 10.3758/s13428-023-02086-5

**Published:** 2023-12-21

**Authors:** Kimberly A. Barchard, James A. Russell

**Affiliations:** 1 Department of Psychology, University of Nevada, Las Vegas, 4505 Maryland Parkway, Las Vegas, NV 89154-5030 USA; 2 Department of Psychology and Neuroscience, Boston College, Chestnut Hill, MA 02467 USA

**Keywords:** Censoring, Missing data, Survival analysis, Limit of detection, Maximum likelihood, Correlation

## Abstract

Data censoring occurs when researchers do not know precise values of data points (e.g., age is 55+ or concentration ≤ .001). Censoring is frequent within psychology but typically unrecognized outside of longitudinal studies. We describe five circumstances when censoring may occur, demonstrate censoring distorts correlations, and discuss how censoring can create spurious factors. Next, we explain how to use R package *lava* to calculate maximum likelihood estimates (Holst and Budtz-Jørgensen *Computational Statistics, 28*(4), 1385–1452, 2013) of correlations between uncensored variables based upon censored variables. Previous research demonstrated these estimates were more accurate than Muthén’s (1984) estimate for one particular model, but no research has systematically examined their accuracy. We therefore conducted a simulation study exploring the effects of the correlation, sample size, and censoring on point and interval estimates of correlations. Based upon 80 cells in which low values of normally distributed variables were censored, we recommend the constrained regression model with Wald confidence intervals. These methods were precise and unbiased unless both variables had 70% censoring and the correlation was large and negative (e.g., −.9), in which case estimates were closer to −1 than they should be. Opposite results would occur if low values of one variable and high values of the other were censored: Estimates would be precise and unbiased unless censoring was extreme and correlations were large and positive. To estimate large correlations accurately, we recommend researchers reduce censoring by using longer longitudinal studies, using scales with more response options, and matching measures to populations to reduce floor and ceiling effects.

**Supplementary Information:**

Supplementary material is available at 10.3758/s13428-023-02086-5.

Censoring occurs when researchers have only partial information about the value of a variable, knowing the value is at least as large as (or no larger than) a given limit of detection. Censoring can distort statistical results and invalidate conclusions (Fox, 2016). A variety of methods exist to correct for this distortion when estimating univariate statistics such as means and standard deviations, often under the title of survival analysis (Allignol & Latouche, 2022) or missing data analysis (Josse et al., 2022). A variety of methods also exist for regression when either the predictor or criterion variable has been censored (Gijbels, 2010). However, only a few methods exist to estimate correlations when both variables are censored, and little is known about their effectiveness. Correlations are used throughout psychology to examine substantive relations, quantify reliability and validity, and conduct multivariate analyses. Therefore, this paper will evaluate a recently developed method of estimating the correlation between two censored variables – a method that has yet to be evaluated systematically.

We begin by describing situations in which censoring may occur. Second, we show the effect of censoring on correlations using both mathematical derivations and simulations and explain how these effects can lead to erroneous multivariate results, such as spurious factors. Third, we explain how to use Holst and Budtz-Jørgensen’s (2013) maximum likelihood method (available in R package *lava*) to estimate the correlation between uncensored variables using the available empirical data from censored variables. Finally, we report a simulation study to evaluate the point and interval estimates obtained using that method.

## When censoring occurs

Missing data can be caused by either censoring or truncation. Censoring occurs when researchers have *partial* information about values falling beyond certain limits; truncation occurs when researchers have *no* information about such values: The data are simply missing. For example, if researchers use the Graduate Record Exams (GRE) to predict graduate school performance, graduate GPAs are likely to suffer from both censoring and truncation (Huitema & Stein, 1993), but for different reasons. Graduate GPAs suffer from censoring because of a ceiling effect: Most graduate students obtain A grades, so these grades do not distinguish between high-scoring students. Graduate GPAs suffer from truncation because of limited admissions: Students who are not admitted have graduate GPAs that are missing.

It is well known that truncation reduces the magnitude of correlations. For example, truncation severely reduces the correlations between GRE scores and graduate school performance (e.g., compare Wao et al., 2016, with correlations of .06 to .11, with Huitema and Stein, 1993, with correlations of .55 to .70). Moreover, corrections for truncation are well known and commonly used (Enders, 2011; Jeličić et al., 2009). Traditional corrections include Pearson’s (1903) correction for restriction of range and Thorndike’s (1949) case 2 correction for direct truncation on the predictor, a correction advocated by Hunter and Schmidt (1990) as a routine part of meta-analysis. Modern approaches to missing data distinguish between data missing at random, completely at random, and not at random (Little & Rubin, 2020), resulting in dozens of parametric and non-parametric methods of correcting for missing data (e.g., Josse et al., 2022).

In contrast, censoring is recognized in some disciplines, but not others. Medical and biological research routinely recognizes the importance of censoring and therefore corrects results to take it into account. For example, van Doremalen et al. (2020) estimated how long SARS-CoV-2 (the virus that causes COVID-19) lives on surfaces by tracking concentrations of the virus over 72 h. Censoring occurred because the virus was still detectable when the study ended. Lauer et al. (2020) estimated the incubation period for the virus: the time from exposure to first symptoms. Censoring occurred because infected people may not know when they were exposed. Tindale et al. (2020) also estimated the incubation period, but addressed the above concern by using cases for whom the exposure date was known.

Within psychology, censoring goes largely unrecognized. Censoring is sometimes recognized in studies that relate psychological variables to medical issues (Hahn et al., 2019; Matt et al., 2004), perhaps because authors became familiar with censoring by reading medical journals. Censoring is also commonly recognized in longitudinal studies (e.g., Ghisletta et al., 2006; Larson et al., 2013; Stice et al., 2000), where psychologists often correct for censoring caused by drop outs or incomplete data. However, in most psychology studies, researchers fail to recognize censoring may have occurred and may have distorted their findings.

In psychology, we contend that censoring occurs in at least five circumstances. First, right censoring occurs when a longitudinal study is tracking the time until an event occurs, and the event is not recorded for some participants. For example, if a marriage study ran for 5 years, and some participants did not marry before the study ended, the researcher knows it was at least 5 years before those participants got married. Time to marriage would be listed as being at least 5 years. However, those participants could have married after 6 years, or after 20, or not at all. Similarly, if a participant left the study after 1 year, the researcher knows it was at least 1 year before that participant married. For that participant, time to marriage would be listed as being at least 1 year. These data are referred to as *right censored* because the right-hand tail of the distribution (which shows high values of the variable) has been obscured, so researchers cannot clearly distinguish between those high values. Right censoring occurs for a variety of outcome variables, such as time to graduation, length of unemployment, and illness duration.

Second, censoring can occur because of difficulties in detecting phenomena. If a behavior occurs very briefly, with low intensity, or under circumstances that make it hard to detect, observers may miss the behavior. For example, if an observer notes a child smiled twice during a five-minute observation period, the child might have smiled when the observer could not see, or the smile might have been brief or small. Thus, right censoring may have occurred because the real number of smiles might be more than two. Conversely, left censoring may occur because measures are only able to detect certain concentrations or frequencies of the thing of interest. For example, a certain level of testosterone must be present in a blood sample before it can be detected (Wang et al., 2014); this is called the limit of detection. When no testosterone is detected, the concentration might be any value below this lower limit. These data are referred to as *left censored* because the left-hand tail of the distribution has been obscured.

Third, censoring can occur because of grouping or rounding. For example, if a respondent selects from among income ranges, researchers know only that income fell between certain limits (e.g., between $30,000 and $39,999). Similarly, if respondents state their age in years or the size of their city in millions, researchers only know that values fall within certain intervals. These data are referred to as *interval* censored.

Fourth, censoring occurs on maximum performance items that are too easy or too hard for some respondents. This can lead to either right or left censoring. For example, imagine a math test contains two problems. The first is easy for grade 5 students, and many students earn the highest possible score. Right censoring occurs because this item fails to distinguish among students earning perfect scores (most of whom know only grade 5 math, but some of whom know grade 6). This is also referred to as a ceiling effect. Conversely, the second problem is difficult for grade 5 students, and many earn a score of 0. Left censoring occurs because this item fails to distinguish among students who scored 0 (most of whom struggle with grade 5 math, but some of whom struggle with grade 4). This is also referred to as a floor effect.

Fifth, censoring can occur because rating scales target one part of a dimension, but respondents are not concentrated there. This can lead to left or right censoring or both at once. Left censoring occurs if a scale is designed to distinguish among people at the high end, but is administered to people outside that range (perhaps respondents vary widely or are concentrated at the low end). For example, imagine people are asked how often they have suicidal thoughts, from never to multiple times per day. If this item were administered to nonclinical populations, left censoring would occur if respondents were not suicidal but nonetheless varied in how depressed they were. Conversely, right censoring occurs if a scale is designed to distinguish among people at the low end of a dimension, but is administered to people in the middle or high end. For example, imagine people are asked how much they agree with the statement, *I avoid crowds.* This item distinguishes between moderate and low levels of extraversion, but not between moderate and high. Finally, both left and right censoring may occur if a scale is designed to distinguish among scores at the middle of a dimension, but respondents include those who are very high and those who are very low. For example, imagine people are asked if they agree, disagree, or neither agree nor disagree with the item, *I am friendly.* Left censoring occurs if people who disagree vary substantially in their friendliness, while right censoring occurs if people who agree vary substantially. Using a larger number of scale points might reduce censoring (e.g., Page et al., 2010). On the other hand, using dichotomous formats (e.g., true/false) may guarantee substantial censoring: With only two response options, people who obtain the same score probably vary substantially.

**Fig. 1:**
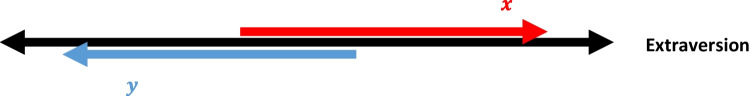
Censoring on positively keyed and negatively keyed items

A special case occurs for rating scales with both positively and negatively keyed items. Censoring is likely to occur because these items are designed to target the two ends of the dimension. Positively keyed items capture high levels of the construct; negatively keyed items, low levels. For example, imagine *X* is the positively keyed item, *I like to go to wild parties*. It distinguishes between moderate and high levels of extraversion, but not between moderate and low levels if all people with low and moderate-to-low levels disagree with this item and obtain the lowest possible score. Conversely, imagine *Y* is the negatively keyed item, *I prefer quiet places.* It distinguishes between moderate and low levels of extraversion, but not between high and moderate-to-high levels if all people with high and moderate-to-high levels disagree and obtain the lowest possible score. Thus, both *X* and *Y* have left censoring (see Fig. 1).

Our examples illustrate that censoring may occur in a wide variety of psychological studies. Moreover, censoring may occur for multiple reasons within the same study, as when repeated testing leads to ceiling effects in longitudinal studies, which also experience participant drop-out (Wang et al., 2008). Thus, psychologists should understand the effects of censoring on their results and take steps to correct for its distortion. This paper describes how censoring effects correlations, explains what corrections can be applied, and evaluates the performance of a recently developed maximum-likelihood correction. We begin by using mathematical derivations and simulations to show the effect of censoring.

## Part 1: The effect of censoring on the correlation

Let *X* and *Y* be two variables that cover the whole of two constructs of interest. Let *x* and *y* be censored versions of these variables. For example, imagine *x* is left censored: Instruments can only detect *X* when it is greater than the limit of detection. If this limit is a concentration of .001, then *x* = *X* when *X* > .001 and *x* = .001 otherwise. If researchers know *x* > .001, they know the exact concentration for *X*. However, if researchers are told *x* is .001, they know only that *X* is .001 or less, not the exact value. Conversely, imagine *y* is right-censored: On a certain questionnaire, the highest observable value for *Y* is an age of 55. Thus, *y* = *Y* when *Y* < 55 and *y* = 55 otherwise. Only if *y* is less than 55 do researchers know the exact value.

**Fig. 2:**
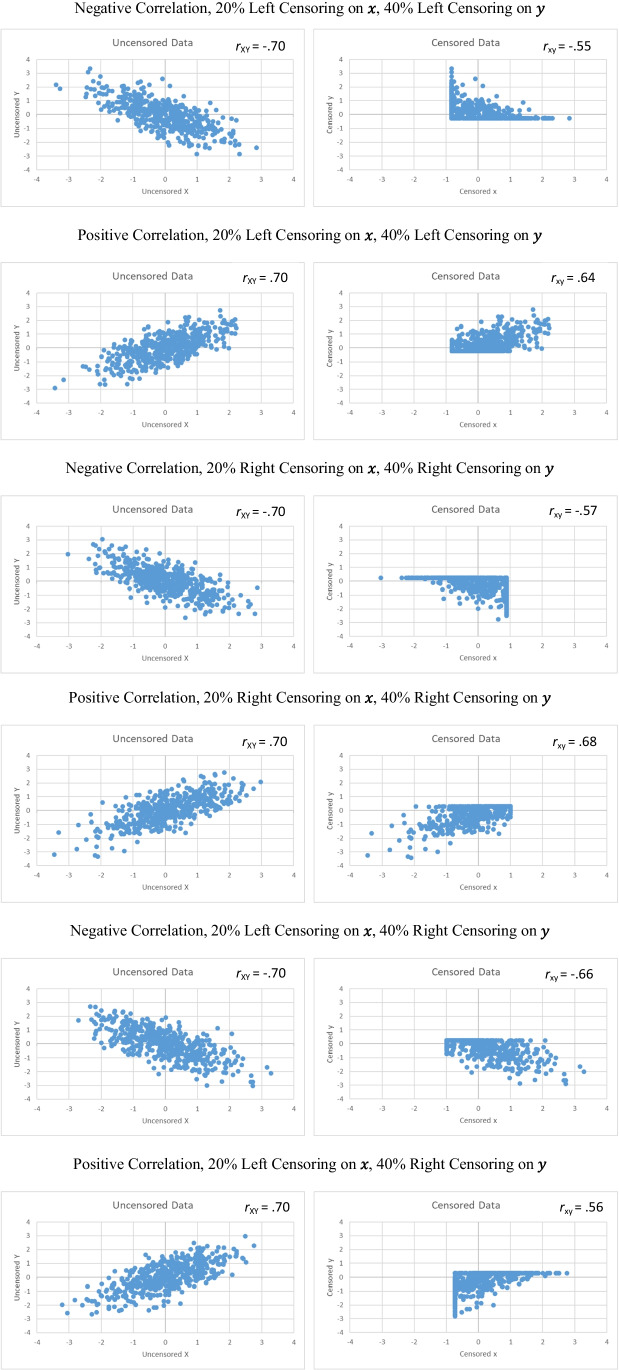
The effect of censoring on correlations. Each panel includes a sample of 500 cases selected from a population with a bivariate standard normal distribution. The left side shows the uncensored data with a correlation of ±.70. The right side shows the censored data in which low (or high) values have been set to the lower (or upper) limit of detection and shows the correlation between these censored scores

When *x* and *y* are censored, their correlation (*ρ*_*xy*_ in the population, *r*_*xy*_ in the sample) is not equal to the correlation between the uncensored variables (*ρ*_*XY*_ in the population, *r*_*XY*_ in the sample). Figure 2 shows the effect of censoring on the correlation in six datasets, each containing 500 cases from a bivariate normal distribution in which *ρ*_*XY*_ =  ± .70. As these examples illustrate, if two variables are censored in the same direction (both left censored or both right censored), their correlations are distorted more when the relation is negative than when it is positive. However, if two variables are censored in opposite directions (one left censored, the other right), their correlations are distorted more when the relation is positive.

This section explores the effect of censoring on correlations. We start by deriving the correlation between *x* and *y* for two special cases: when they each cover the whole of their dimensions (no censoring) or precisely half (50% censoring). This second derivation uses left censoring on both variables. Left censoring and right censoring are mirror images. Formulas and programs designed for one can be used for the other by taking the inverse of the data (i.e., multiplying the data by −1). Therefore, we use the derivation for left censoring to infer the effect of right censoring on both variables or left censoring on one and right censoring on the other. After considering the special cases of 0% and 50% censoring, we use simulations to estimate the correlation between *x* and *y* for any degree of left or right censoring on each of *x* and *y*. Finally, we examine the implications of these results for factor analyses based upon the positive and negative correlations caused by positively and negatively keyed items.

In all these derivations and simulations, the correlation between the censored variables depends upon the correlation between *X* and *Y*, which in turn depends upon the angle between *X* and *Y*. When there is censoring, the correlation also depends upon the degree of censoring on *x* and *y*, the type of censoring (left or right), and the bivariate distribution of *X* and *Y*. To provide a sense of the effect censoring can have even for nearly ideal data, we will assume *X* and *Y* have a continuous bivariate normal distribution.

### No censoring

If *x* and *y* each have 0% censoring, then *x* and *y* cover the whole of *X* and *Y*, respectively, which means *x* = *X* and *y* = *Y* for all values of *X* and *Y*. Therefore, *ρ*_*xy*_ = *ρ*_XY_.

The values of these correlations can be calculated based upon the angle between *X* and *Y*. By the law of cosines, the correlation between two zero-centered variables equals the cosine of the angle between them (Rodgers & Nicewander, 1988). Therefore, if *X* and *Y* are zero-centered,

1 $${\rho}_{xy}={\rho}_{XY}=\cos \theta,$$

where *θ* is the angle between *X* and *Y*. For example, when θ = 0°, *x* = *y* and *ρ*_*xy*_ = 1. When θ = 180°, *x* and *y* are exact opposites and *ρ*_*xy*_ =  − 1. When θ = 90°, *x* and *y* are independent and *ρ*_*xy*_ = 0. When θ = 135°, *x* and *y* angle away from each other and *ρ*_*xy*_ =  − .707.

### 50% censoring

The effect of censoring on correlations depends upon whether both variables have left censoring, both have right, or one has left and the other right. We begin by considering left censoring.

If *x* and *y* each have 50% left censoring, they cover the top halves of *X* and *Y*, respectively. In this case, *ρ*_*xy*_ does not usually equal *ρ*_*XY*_ and we need to calculate it. However, correlations depend upon the distributions of the variables. Therefore, let us assume *X* and *Y* have a bivariate standard normal distribution (implying *x* = *X* for *X* > 0, and *x* = 0 otherwise; *y* = *Y* for *Y* > 0, and *y* = 0 otherwise).

*ρ*_*xy*_ can be calculated using the general equation for a correlation as a function of expected values:

2 $${\rho}_{xy}=\frac{E\left[ xy\right]-E\left[x\right]E\left[y\right]}{{\left\{\left[E\left[{x}^2\right]-{\left(E\left[x\right]\right)}^2\right]\left[E\left[{y}^2\right]-{\left(E\left[y\right]\right)}^2\right]\right\}}^{1/2}}.$$

As noted by Carroll (2000; Russell & Carroll, 1999), the positive values of *x* and *y* have half normal distributions, so that $E\left[x\right]=E\left[y\right]={\left(\frac{1}{2\pi}\right)}^{1/2}$ and $E\left[{x}^2\right]=E\left[{y}^2\right]=\frac{1}{2}$. Thus^1^,

3 $${\rho}_{xy}=\frac{E\left[ xy\right]-\frac{1}{2\pi }}{\frac{1}{2}-\frac{1}{2\pi }}.$$

As noted by Carroll (2000), *E*[*xy*] can be calculated based upon the bivariate density function for the standard normal distribution:

- $$E\left[ xy\right]={\int}_0^{\infty }{\int}_0^{\infty } XYf\left(X,Y\right) dXdY$$, where
- $$f\left(X,Y\right)=\frac{e^{-.5\left({X}^2+{Y}^2-2{ XY\rho}_{XY}^2\right){\left(1-{\rho}_{XY}^2\right)}^{-1}}}{2\pi {\left(1-{\rho}_{XY}^2\right)}^{1/2}}$$, and
- *ρ*_*XY*_ = cos(θ).

Therefore^1^,

4 $${\rho}_{xy}=\frac{\left[{\int}_0^{\infty }{\int}_0^{\infty } XYf\left(X,Y\right) dXdY\right]-\left[\frac{1}{2\pi}\right]}{\frac{1}{2}-\frac{1}{2\pi }}.$$

Formula 4 can be used to determine how the correlation changes when *x* and *y* each have 50% left censoring. For example, when θ = 0°, *X* = *Y* for every value of *X* and *Y* (and hence every value of *x* and *y*) and so censoring does not change the correlation: *ρ*_*XY*_ = *ρ*_*xy*_ = 1. When θ = 90°, *X* and *Y* are independent and censoring again has no effect: *ρ*_*XY*_ = *ρ*_*xy*_ = 0, as expected. In contrast, when θ = 180°, *X* and *Y* are exact opposites and censoring changes the correlation dramatically: *ρ*_*XY*_ =  − 1, while *ρ*_*xy*_ =  − .467. Finally, when θ = 135°, censoring has an intermediate effect: *ρ*_*XY*_ =  − .707, while *ρ*_*xy*_ =  − .396.

The above calculations assumed 50% *left* censoring on both *x* and *y*. These same *ρ*_*xy*_ would occur if there was 50% *right* censoring on both. If instead there was 50% left censoring on one variable and 50% right censoring on the other, the reverse would occur. When θ = 180°, censoring has no effect: *ρ*_*XY*_ = *ρ*_*xy*_ =  − 1. When θ = 0°, censoring has a dramatic effect: *ρ*_*XY*_ = 1, while *ρ*_*xy*_ = .467.

### Arbitrary degrees of censoring

Rarely will two empirical variables each have precisely 50% censoring. Therefore, we created an Excel file to quickly estimate *ρ*_*xy*_ when variables have arbitrary degrees of censoring: CensorCorr. CensorCorr (Barchard, 2022) randomly generates scores on *X* and *Y* for a sample of 500,000 cases drawn from a population in which *X* and *Y* have a bivariate normal distribution with the specified value of *ρ*_*XY*_. Next, CensorCorr calculates scores for *x* and *y* by applying the user-specified degrees of left and right censoring. Finally, CensorCorr calculates the sample correlation between *x* and *y*. Because of the large sample size, this sample correlation (*r*_*xy*_) is a good estimate of the population correlation of interest (*ρ*_*xy*_). For example, when we specified *ρ*_*XY*_ =  − 1 and *x* and *y* each have 50% left censoring, CensorCorr estimated *ρ*_*xy*_ =  − .466, which is close to the −.467 value derived earlier.

CensorCorr can help researchers understand the effects of censoring. For example, if researchers find that one variable has 25% floor effects and the other 45%, a few quick trials show that an obtained *r*_*xy*_ = −.38 is consistent with the somewhat larger *ρ*_*XY*_ = −.50. More generally, researchers can use CensorCorr to explore how different kinds of censoring affects correlations and to learn how censoring affects scatterplots and histograms (so they can recognize possible cases of censoring in their own and others’ work).

**Fig. 3:**
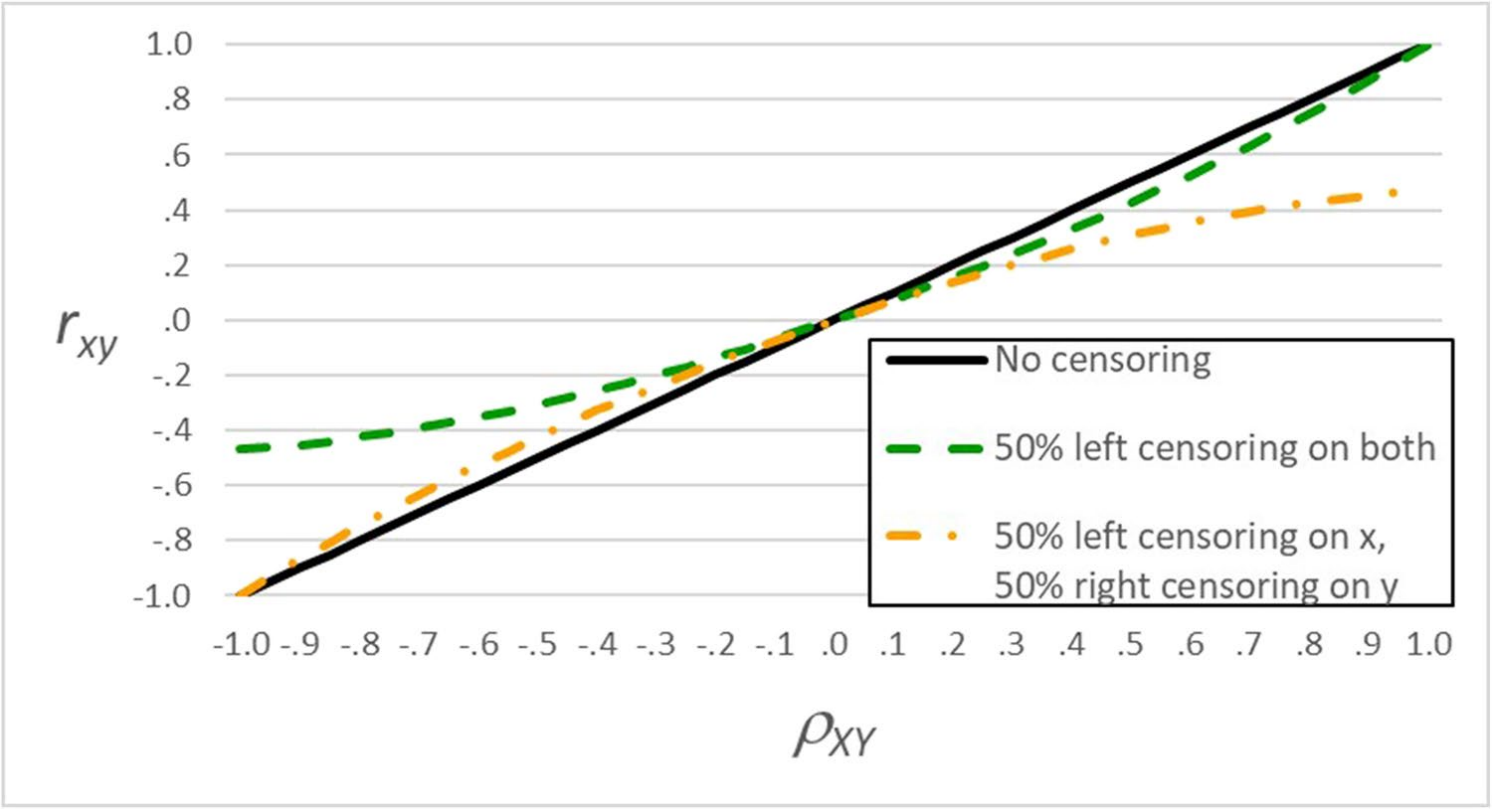
The correlation between censored variables (*r*_*xy*_) as a function of the correlation between uncensored variables (*ρ*_*XY*_) and censoring pattern. The green dashed line shows censoring has a larger effect on negative correlations than positive correlations when both variables are left censored. Conversely, the orange dash-dotted line shows censoring has a larger effect on positive correlations than negative correlations when one variable is left censored and the other is right censored. The correlations between censored variables (*r*_xy_) were calculated using CensorCorr (Barchard, 2022), which first simulated samples of 500,000 cases from populations with bivariate normal distributions with the specified values of *ρ*_*XY*_ and then applied the specified censoring

Using CensorCorr, we created Fig. 3 to show the relation between *ρ*_*XY*_ and *ρ*_*xy*_ for two patterns of censoring. When *x* and *y* both have 50% left censoring, the green dashed line shows negative correlations are affected more than positive ones: *ρ*_*xy*_ ranges from –.467 to 1. In contrast, when *x* has 50% left censoring and *y* has 50% right, the orange dash-dotted line shows positive correlations are affected more than negative ones: *ρ*_*xy*_ ranges from –1 to .467.

The green dashed line in Fig. 3 reveals an important asymmetry: If two uncensored variables have a perfect negative relation (*A* =  − *B*), but both have left censoring, their correlations with a third variable will not be inverses of each other. For example, imagine variables *A* and *C* are separated by 45°: *ρ*_*AC*_ = .707 and *ρ*_*BC*_ =  − .707. If *a*, *b*, and *c* each have 50% left censoring, the dashed green line shows^2^*ρ*_*ac*_ is estimated as .651, while *ρ*_*bc*_ is estimated as −.385. Clearly, .651 and −.385 are not symmetric.

#### Implications for factor analysis

The asymmetric effect of censoring on positive and negative correlations can explain a common finding in factor analytic studies: Negatively keyed items frequently load on separate factors from positively keyed items (Schmitt & Stults, 1985). Whether or not separate factors occur depends upon how items are written.

Positively and negatively keyed items are typically written to measure opposite ends of a dimension. To measure the full breadth of a construct, items often present extreme statements and respondents are asked how much they agree with them. For example, Goldberg’s (1992) Extraversion Scale includes the following items: *Am the life of the party* and *Am a very private person*. Because these are extreme statements, people who indicate these statements are *very inaccurate* are likely to be heterogeneous. For example, someone who indicates that *Am the life of the party* is *very inaccurate* may have either a moderate level of extraversion or a low level. These people all receive the lowest score on this item (1 = *very inaccurate*), and so scores are left censored. Similarly, people who indicate that *Am a very private person* is *very inaccurate* may have either a moderate level of extraversion or a high level. These people all receive the lowest score, despite varying on the dimension of interest, and so these scores are also left censored.

When all items are left censored, the green dashed line in Fig. 3 shows each item correlates more highly with items keyed in the same direction. Positively keyed items correlate more highly with other positively keyed items; negatively keyed items correlate more highly with other negatively keyed items. The result: separate factors for positively and negatively keyed items.

On the other hand, sometimes negatively keyed items simply negate positively keyed items. For example, the Goldberg’s (1992) Emotional Stability Scale includes the items *Seldom feel blue* and *Often feel blue*. And Hofstee et al.’s (1992) Conscientiousness Scale includes the items *Like to plan ahead* and *Do not plan ahead*. Pairs of items such as these are likely to be censored in opposite directions: Left censoring on positively keyed items is associated with right censoring on negatively keyed items. For example, people who indicate that *Like to plan ahead* is *very inaccurate* may have moderate or low levels of conscientiousness (leading to left censoring on this item) and people who indicate that *Do not plan ahead* is *very accurate* may also have moderate to low levels of conscientiousness (leading to right censoring on this item).

When one variable has left censoring and the other has right, the orange dash-dotted line in Fig. 3 shows the effect of that censoring on their correlation. Censoring reduces the positive correlations between items keyed in the same direction (note that the orange dash-dotted line moves away from the black solid line in the upper right-hand corner of Fig. 3, showing censoring has a strong effect on positive correlations), thus reducing the factor loadings for those items. Censoring has little effect on the negative correlations between items keyed in opposite directions (note that the orange dash-dotted line is close to the black solid line in the lower left-hand corner of Fig. 3, showing censoring has little effect on negative correlations); therefore, negatively keyed items will load on the same factor as the positively keyed items. Thus, when negatively keyed items simply negate positively keyed items, all items will tend to load on a single factor.

In summary, different strategies for writing negatively keyed items can lead to different patterns of censoring, and those different patterns of censoring can explain why negatively keyed items sometimes load on separate factors from the positively keyed items and sometimes load on the same factor. When negatively keyed items clearly anchor the negative end of a dimension, they will tend to form a separate factor. When negatively keyed items simply negate positively keyed items, they will tend to load on the same factor.

Frequently, psychometricians use factor analyses to determine which items to include on scales. When negatively keyed items fail to load on the same factor as positively keyed items, they are frequently removed from the scale. Using this strategy, psychometricians will tend to retain items that simply negate the positive end of a dimension (e.g., *Do not plan ahead*) and remove items that label the negative end of a dimension clearly (e.g., *Am often late to work*). This is unfortunate. This former type of item is poorly written (in general, it is best to avoid negatives in item stems, especially if the response scale includes negative words such as *false*, *disagree,* or *inaccurate*; Lenzner & Menold, 2016), and these items provide little or no information beyond the original positively keyed items. Therefore, taking into account censoring can help psychometricians better measure the full breadth of their constructs and retain items that better capture the low ends of their dimensions.

## Part 2: Estimating the correlation between uncensored variables

The first part of our paper showed censoring distorts the relations between variables. Fortunately, analytic methods have been created to estimate relations when variables have been censored. Indeed, dozens of methods are available under the names of censored data analysis, survival analysis (e.g., Allignol & Latouche, 2022), and missing data analysis (e.g., Josse et al., 2022). However, the vast majority – for example, Cox (1972) regression, Kaplan–Meier (1958) procedure, mixture models (Zeller et al., 2019), tobit regression (Tobin, 1958), two-part models (Duan et al., 1983), Gibbs sampling (Gilks & Wild, 1992), and robust linear regression on order statistics (Lee & Helsel, 2005) – cannot be used to estimate correlations among censored variables, because they allow censoring on either the dependent variable or the independent variable(s), but not both (Shoari & Dubé, 2018). To estimate the correlation between two uncensored variables, we need a method that allows censoring on both *x* and *y*.

Several methods estimate bivariate correlations when both variables have been censored (Chen et al., 2013; Hoffman & Johnson, 2015; Holst & Budtz-Jørgensen, 2013; Li et al., 2018; Newton & Rudel, 2007; Pesonen et al., 2015; Wang et al., 2019). One of these methods has been implemented in a software package – the R package *lava* (Holst, 2020a) – that can also accommodate multivariate models^3^, such as factor analyses, path analyses, longitudinal models, and other structural equation models, and hence has greater potential impact on applied data analysis. Moreover, *lava* is freely available under General Public License−3 (Free Software Foundation, 2007), and so users can see exactly how the statistics are calculated and can develop extensions as desired (GitHub, n.d.). This makes *lava* a transparent and flexible research tool. Therefore, this current study will examine the effectiveness of *lava*.

*Lava* provides maximum likelihood (ML) estimators for the covariances between uncensored variables using an extension of tobit regression and assuming the uncensored variables have a multivariate normal distribution (see Holst & Budtz-Jørgensen, 2013, for the statistical theory and Holst, 2020c, for the source code). The initial work on this ML method is promising. Holst and Budtz-Jørgensen (2013) provided the mathematical derivations, explained how to calculate the estimates using *lava*, and demonstrated the calculations with brain imaging data. Holst et al. (2015) provided a second demonstration, applying the *lava* package with brain imaging and personality data. They also presented a simulation study showing their estimates are less biased and more precise (i.e., had a smaller mean square error) than the limited information estimator^4^ proposed by Muthén (1984) when estimating a complex structural equation model. However, this simulation examined only a single model (containing a binary outcome variable and five normally distributed variables that had no censoring, with a single set of beta-weights to represent the relations between these variables) with a single sample size (500). No research has systematically examined the accuracy of *lava* estimates with censored data, not even for the simplest estimation task: individual correlations between normally distributed variables that have been censored. Therefore, this current research will examine the accuracy of *lava* in estimating correlations among bivariate normal variables with a variety of sample sizes and censoring patterns.

Within *lava*, the *lvm* function is used to specify the model being tested. Two models can be used to estimate a correlation. The first is a correlation model (X~~Y). This is the obvious model to use when researchers want to estimate a correlation. The second is a regression model (~X+Y). While it may seem counter-intuitive to use regression to estimate a simple correlation, using a regression model in *lava* allows us to constrain the solution to ensure estimated variances and covariances form a positive definite matrix. This constraint prevents estimates that are outside the allowable range for a correlation (−1 to +1) and increases the chance *lava* will converge upon a solution (Holst, personal communication, April 2020).

Using those models, the *estimate* function provides both a point estimate and a 95% confidence interval for the correlation. By default, the *estimate* function calculates Wald confidence intervals. The Wald confidence interval is the one that results from adding and subtracting 1.96 times the standard error. However, Wald confidence intervals may not be appropriate if *ρ*_*XY*_ is close to ±1 (as would be the case, for example, if *X* and *Y* were opposites). If the correlation model was used, the confidence intervals could extend past ±1; if the regression model was used, constraining the confidence interval to be within the allowable bounds might affect its location or width. Therefore, researchers may wish to use profile confidence intervals, instead. The end points of the 95% profile confidence intervals are the 2.5th and 97.5th percentiles of the log-likelihood function. Profile confidence intervals are available using the *confint* function.

Using *lava* to calculate point and interval estimates for correlations among uncensored variables is simple. However, the necessary code is not readily apparent from the two published papers or the *lava* manual (Holst, 2020a). Therefore, the appendix provides detailed instructions for specifying the models and calculating point and interval estimates of the correlation.

## Part 3: Evaluating the accuracy of point and interval estimates of the correlation between uncensored variables

We conducted a simulation study to evaluate the accuracy and precision of *lava* estimates of the correlation between uncensored variables. We conducted this study using datasets where both variables had left censoring, then we inferred the effect when both variables have right censoring or one variable has left censoring and the other right. We used *lava* version 1.6.8 (Holst, 2020a) and *mets* version 1.2.8 (Holst, 2020b), which we downloaded from GitHub (Holst, 2020c) on June 4, 2020. Current and previous versions of *lava* are available at https://CRAN.R-project.org/package=lava The R code for our simulation study is available under supplementary materials.

### Method

To identify the circumstances leading to strong and weak performance for the estimates, we simulated multivariate normal data sets that have a wide range of population correlations, a wide variety of censoring patterns, and both moderate and large sample sizes. We examined small correlations (*ρ*_*XY*_ =  ± .3), large correlations (*ρ*_*XY*_ =  ± .7), very large correlations as might be seen when evaluating reliability (*ρ*_*XY*_ =  ± .9), and perfect correlations (*ρ*_*XY*_ =  ± 1). We examined five patterns of censoring on *x* and *y*, ranging from moderate censoring to severe: (a) 30% left censoring on both *x* and *y*, (b) 50% on both, (c) 70% on both, (d) 0% on *x* and 50% on *y*, and (e) 30% on *x* and 70% on *y*. Finally, censored data analysis is primarily a large sample technique; nonetheless, we used both large (500) and medium (200) samples. We examined every possible combination of *ρ*_*XY*_, sample size, and censoring pattern, resulting in 80 cells. In addition, to allow significance tests of possible four-way interactions between estimation method, *ρ*_*XY*_, sample size, and censoring pattern, we ran two replications of each cell.

For each replication of each cell, we ran 1000 trials. We selected 1000 because it is the most common number of simulations in Monte Carlo studies (Morris et al., 2019) and would allow precise estimates of the statistics used to evaluate the estimates (e.g., bias, coverage, etc., described below). However, because we conducted two replications of each cell, our summary statistics are based upon 2000 trials, and the results from the two replications can be compared to evaluate the precision of each estimate (see Supplementary Table 1 for the results of each replication of each cell).

Within each trial, we generated a random set of data (using package *mvrnorm*, function *rmvnorm*) for which *X* and *Y* had a bivariate normal distribution with the desired correlation. Then we left censored *x* and *y*; for example, if *x* had 30% censoring, we replaced the lowest 30% of the scores with the 30th percentile. We provided the *x* and *y* data to *lava* to estimate the correlation between *X* and *Y* (*ρ*_*XY*_) using both the correlation and regression models. Finally, we asked *lava* to provide three 95% confidence intervals for that correlation: the Wald confidence interval based upon the correlation model, the Wald confidence interval based upon the regression model, and the profile confidence interval based upon the regression model.

To evaluate the quality of the estimate ${\hat{\rho}}_{XY}$, we summarized the 2000 trials for each cell by calculating six statistics. The mean estimated correlation (${\hat{\rho}}_{XY}$) should be close to the actual correlation (*ρ*_*XY*_). The standard deviation of those estimates should be small (ideally 0). The minimum and maximum values of ${\hat{\rho}}_{XY}$ should be close to *ρ*_*XY*_. The bias (the mean difference between ${\hat{\rho}}_{XY}$ and *ρ*_*XY*_) should be low (ideally 0). The root mean square error (RMSE; the square root of the mean of the squared differences between ${\hat{\rho}}_{XY}$ and *ρ*_*XY*_) should also be low.

To evaluate the quality of the confidence intervals for *ρ*_*XY*_, we calculated seven statistics. The proportion of trials converging upon estimates for the intervals (i.e., the confidence interval function providing a numerical result rather than a blank space or an error message) should be high (ideally 1.00). The midpoint of the intervals should be close to *ρ*_*XY*_ when averaged across trials. The proportion of the 95% intervals including *ρ*_*XY*_ should be high (for most cells, this proportion should be near .95; however, for cells where the confidence interval width is 0 for all trials^5^, this proportion should be 1). The maximum, mean, and median widths of the intervals across all trials should all be small (to indicate the intervals provide precise information about *ρ*_*XY*_), and ideally the maximum should be similar to the median (to indicate the intervals are performing consistently). These widths can also be used to understand why intervals sometimes capture *ρ*_*XY*_ less often or more often than desired. Finally, the proportion of intervals having widths greater than or equal to 2 should be low (ideally 0).

In this study, profile intervals cannot have end points outside the range [−1, +1] because of boundaries set on the *z* values, so cannot have widths greater than or equal to 2. Regression model Wald intervals might similarly avoid end points beyond ±1 because these models are constrained to produce positive definite estimates of the variance-covariance matrices. However, even a width of 2 is catastrophic. Intervals ranging from −1 to +1 provide no information about *ρ*_*XY*_. Worse yet, because there are no constraints on correlation model Wald intervals, estimates outside that range (e.g., an interval ranging from −5 to +5) might be possible. Thus, the maximum width of the intervals and the proportion of the intervals whose widths are greater than 2 provide critical information about the usefulness of the confidence interval methods.

To summarize results across the 80 cells, we conducted between-within ANOVAs and drew graphs of significant interactions. For all analyses, the within-cell factor was the estimation method, and the between-cell factors were the three design factors: *ρ*_*XY*_, sample size, and pattern of censoring on *x* and *y*. To evaluate the quality of the two point estimates, we used three of the six outcome variables: proportion of trials converging upon estimates, bias, and RMSE. To evaluate the quality of the three interval estimates, we used three of the seven outcome variables: proportion of trials converging upon estimates, proportion of intervals including *ρ*_*XY*_, and mean interval width.

### Results

The results for all 13 outcome variables for the two replications of each of the 80 cells are given in Supplemental Table 1. For each cell, this table shows the two replications provided very similar results, indicating the number of trials was sufficient.

#### Point estimates of ρ_XY_

*Lava* estimates of *ρ*_*XY*_ worked remarkably well. Both the correlation and regression models usually provided reasonable estimates of *ρ*_*XY*_. However, when there was severe censoring and *ρ*_*XY*_ was large and negative, both models were somewhat biased. Additionally, when *ρ*_*XY*_ =  ± 1, only the regression model consistently provided reasonable estimates of *ρ*_*XY*_.

##### Proportion of trials that converged upon estimates

The regression model converged upon reasonable estimates of *ρ*_*XY*_ in 100% of the trials for every cell. However, the correlation model failed to converge or converged upon unreasonable estimates in some trials in six cells (see Table 1). In all six, *ρ*_*XY*_ was a perfect correlation (±1) and there was unequal censoring on *x* and *y*. In three of these cells, the correlation model sometimes converged upon an invalid estimate of *ρ*_*XY*_ (outside the allowable range of −1 to +1); moreover, these estimates were positive when the true value of *ρ*_*XY*_ was negative or vice versa. Thus, the correlation model occasionally leads to invalid estimates and lack of convergence, while the regression model had no such problems.

**Table 1:** Proportion of trails where correlation model failed to converge upon an estimate of ρ_XY_

*ρ*_*XY*_	Censoring	Sample size	Proportion failing to converge	Proportion providing invalid estimates	Invalid estimates
−1	0% *x* 50% *y*	200	1 / 2000	1 / 2000	1.14
−1	0% *x* 50% *y*	500	1 / 2000	1 / 2000	1.20
1	0% *x* 50% *y*	200	2 / 2000	1 / 2000	−1.03
1	0% *x* 50% *y*	500	1 / 2000		
1	30% *x* 70% *y*	200	20 / 2000		
1	30% *x* 70% *y*	500	1 / 2000		

*Note.* The regression model converged upon a valid estimate of *ρ*_*XY*_ in 100% of the trials in every cell examined. However, in these six cells, in a few trials, the correlation model did not converge. Moreover, in three trials in these cells, the correlation model converged upon an estimate, but the estimate was outside the allowable range for a correlation (−1, 1).

##### Bias

**Fig. 4:**
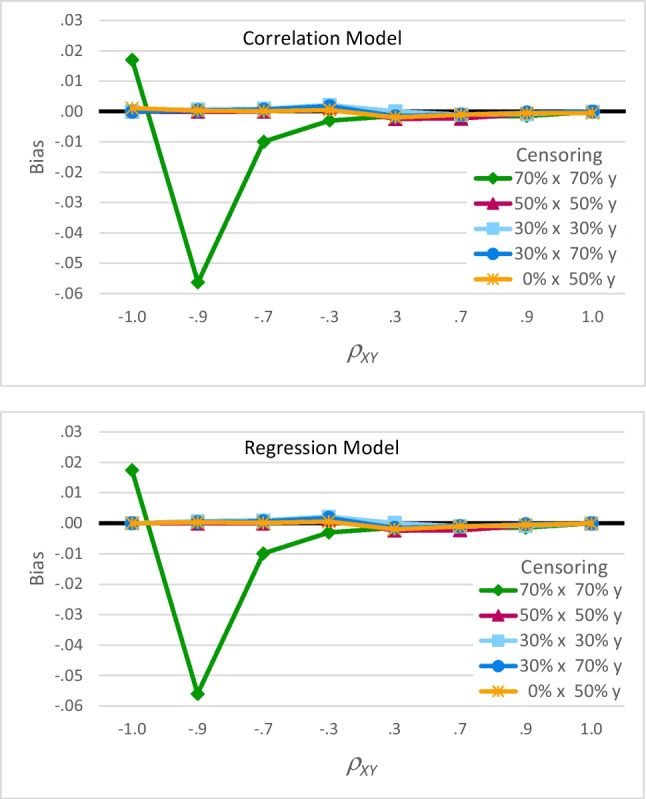
Effects of *ρ*_*XY*_ and censoring pattern on bias. Bias (the mean difference between ${\hat{\rho}}_{XY}$ and ${{\rho}}_{XY}$) was nearly identical for the correlation model in the top panel and the regression model in the bottom panel. For both models, the green diamond-marked line shows ${\hat{\rho}}_{XY}$ was biased for large negative values of *ρ*_*XY*_ when there is 70% censoring on both * x* and *y*. The other lines show ${\hat{\rho}}_{XY}$ was unbiased for other patterns of censoring. In this figure, bias is averaged across the two sample sizes

The correlation and regression models were similar in terms of bias. This similarity can be seen by comparing the top (correlation) and bottom (regression) panels in Fig. 4, which show bias for each value of *ρ*_*XY*_ and each pattern of censoring. Although there was a significant three-way interaction of model, *ρ*_*XY*_, and censoring pattern, *F*(28, 80) = 2.25, *p* = .003, partial eta-squared = .44, the differences in bias between the two models were trivial (always less than .002). Relatedly, this figure shows the *average* bias across the two sample sizes because of the non-significant four-way interaction, *F*(28, 80) = .309, *p* = 1.00, partial eta-squared = .098.

For both models, Fig. 4 clearly shows the two-way interaction of *ρ*_*XY*_ and censoring pattern, *F*(28, 80) = 178.99, *p* < .001, partial eta-squared = .98. Neither model was biased if *ρ*_*XY*_ was positive (the right-hand sides of both panels). Moreover, neither model was biased if *ρ*_*XY*_ was negative and there was no more than moderate censoring (most of the lines on the left-hand sides). However, when *ρ*_*XY*_ was large and negative and both *x* and *y* had 70% censoring (the green diamond-marked lines), both models produced biased estimates. Specifically, when *ρ*_*XY*_ =  − 1 (on the far-left side), ${\hat{\rho}}_{XY}$ values were about .017 further from −1 than they should be (near −.983), and when *ρ*_*XY*_ =  − .9, ${\hat{\rho}}_{XY}$ values were about .055 closer to −1 than they should be (near −.955).

**Fig. 5:**
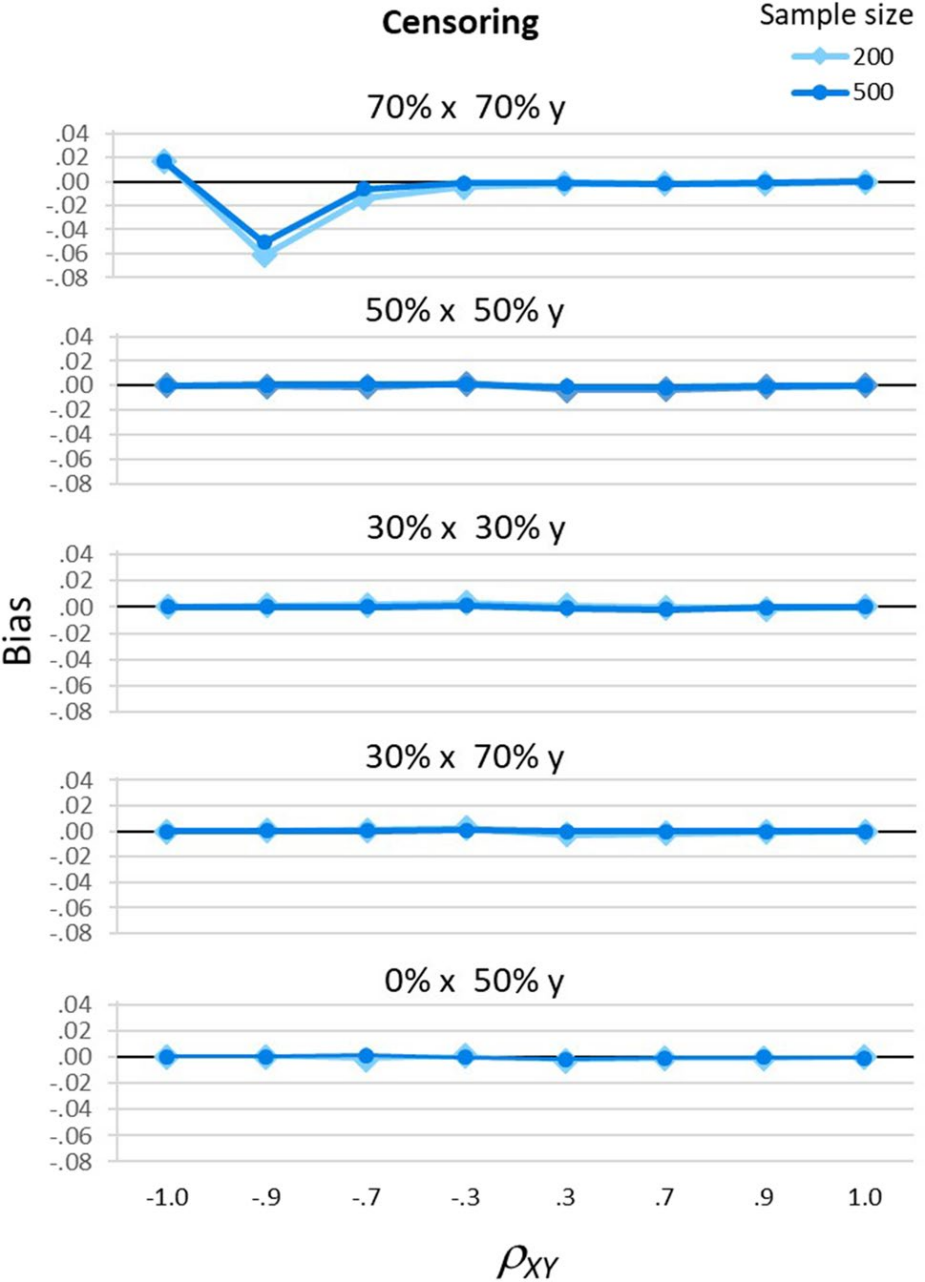
Effects of *ρ*_*XY*_, sample size, and censoring pattern on bias. Bias (the mean difference between ${\hat{\rho}}_{XY}$ and ${{\rho}}_{XY}$) is shown for the five patterns of censoring. When there was 70% censoring on both *x* and *y*, the top panel shows ${\hat{\rho}}_{XY}$ was biased for large negative correlations, but bias decreased when sample size increased from 200 (light diamond-marked lines) to 500 (dark circle-marked lines). For other censoring patterns, the remaining panels show ${\hat{\rho}}_{XY}$ was unbiased for both sample sizes. In this figure, bias is averaged across the correlation and regression models

When bias occurred, increasing the sample size reduced that bias only slightly. Figure 5 shows the significant three-way interaction of sample size, *ρ*_*XY*_, and censoring pattern on mean bias across the two models, *F*(28, 80) = 2.22, *p* = .003, partial eta-squared = .44. This figure shows again that ${\hat{\rho}}_{XY}$ was not biased when *ρ*_*XY*_ was positive (the right-hand sides of each panel) or when *ρ*_*XY*_ was negative with only small to moderate censoring (the middle and left-hand sides of most panels), but that it was biased when *ρ*_*XY*_ was large and negative and both *x* and *y* had 70% censoring (the top panel). These biases decreased as sample size increased; however, the dark circle-marked line for *n* = 500 is only slightly closer to a bias of 0 than the light diamond-marked line for *n* = 200. For example, when *ρ*_*XY*_ =  − .9, bias decreased from −.06 for *n* = 200 to −.05 for *n* = 500, a difference of only .01^6^.

##### RMSE

**Fig. 6:**
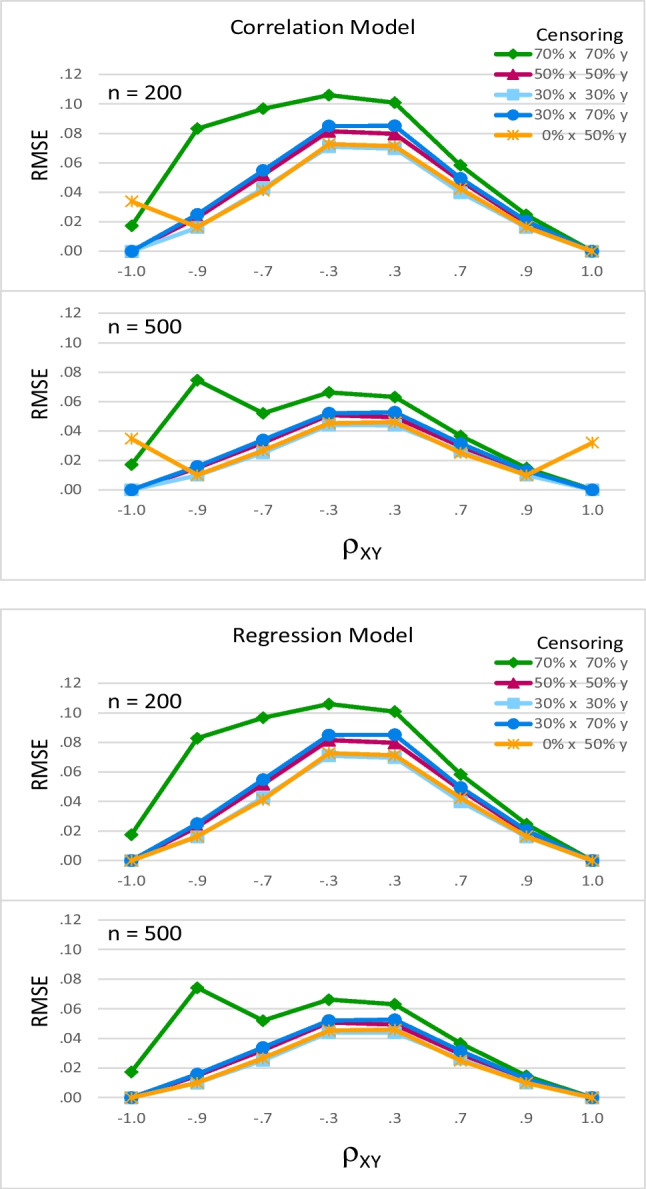
Effects of model*, ρ*_*XY*_, sample size, and censoring pattern on root mean square error. Root mean square error (RMSE) was small for most cells. RMSE was higher when *ρ*_*XY*_ was smaller and censoring was higher. RMSE decreased as sample size increased. However, when *ρ*_*XY *_= –.9 and there was 70% censoring on both x and y (green diamond-marked lines), RMSE was almost as high for *n *= 500 as it was for *n *= 200. Additionally, when *ρ*_*XY *_= ±1, RMSE was 0 or near 0 unless there was 0% censoring on *x* and 50% on *y  *(orange star-marked lines); in those cells, the correlation model occasionally provided invalid estimates of *ρ*_*XY*_, which noticeably increased RMSE

For most combinations of factors, RMSE was similar for the correlation and regression models. For both models, RMSE was small for most cells, indicating estimates were fairly precise. However, *ρ*_*XY*_ interacted with sample size, *F*(7, 80) = 40.56, *p* < .001, partial eta-squared = .780, and censoring pattern, *F*(28, 80) = 17.77, *p* < .001, partial eta-squared = .861. Figure 6 shows RMSE was higher for smaller values of *ρ*_*XY*_ (in the center of each graph) and more censoring (the darker lines) and decreased as sample size increased (the bottom portion of each panel). Notably, sample size interacted with censoring pattern, *F*(4, 80) = 5.39, *p* < .001, partial eta-squared = .212. When *ρ*_*XY*_ =  − .9 and there was censoring of 70% on both *x* and *y* (the green diamond-marked lines in each panel), RMSE was almost as large for *n* = 500 as it was for *n* = 200.

Model interacted with censoring pattern, *F*(4, 80) = 2.99, *p* = .024, partial eta-squared = .130. Specifically, for two combinations of factors, the regression model was superior to the correlation model in terms of RMSE. When *ρ*_*XY*_ = 1 or −1, RMSE was 0 or near 0 unless there was 0% censoring on *x* and 50% on *y* (orange star-marked lines). In those cells, the correlation model occasionally provided invalid estimates of *ρ*_*XY*_, which noticeably increased RMSE.

#### Interval estimates of *ρ*_*XY*_

We examined three confidence intervals for *ρ*_*XY*_: the Wald confidence interval based upon the correlation model (correlation-Wald), the Wald confidence interval based upon the regression model (regression-Wald), and the profile confidence interval based upon the regression model (regression-profile). The regression-Wald method was superior for the kinds of data that are likely to be encountered in empirical research.

##### Proportion of trials that converged upon confidence interval estimates

**Fig. 7:**
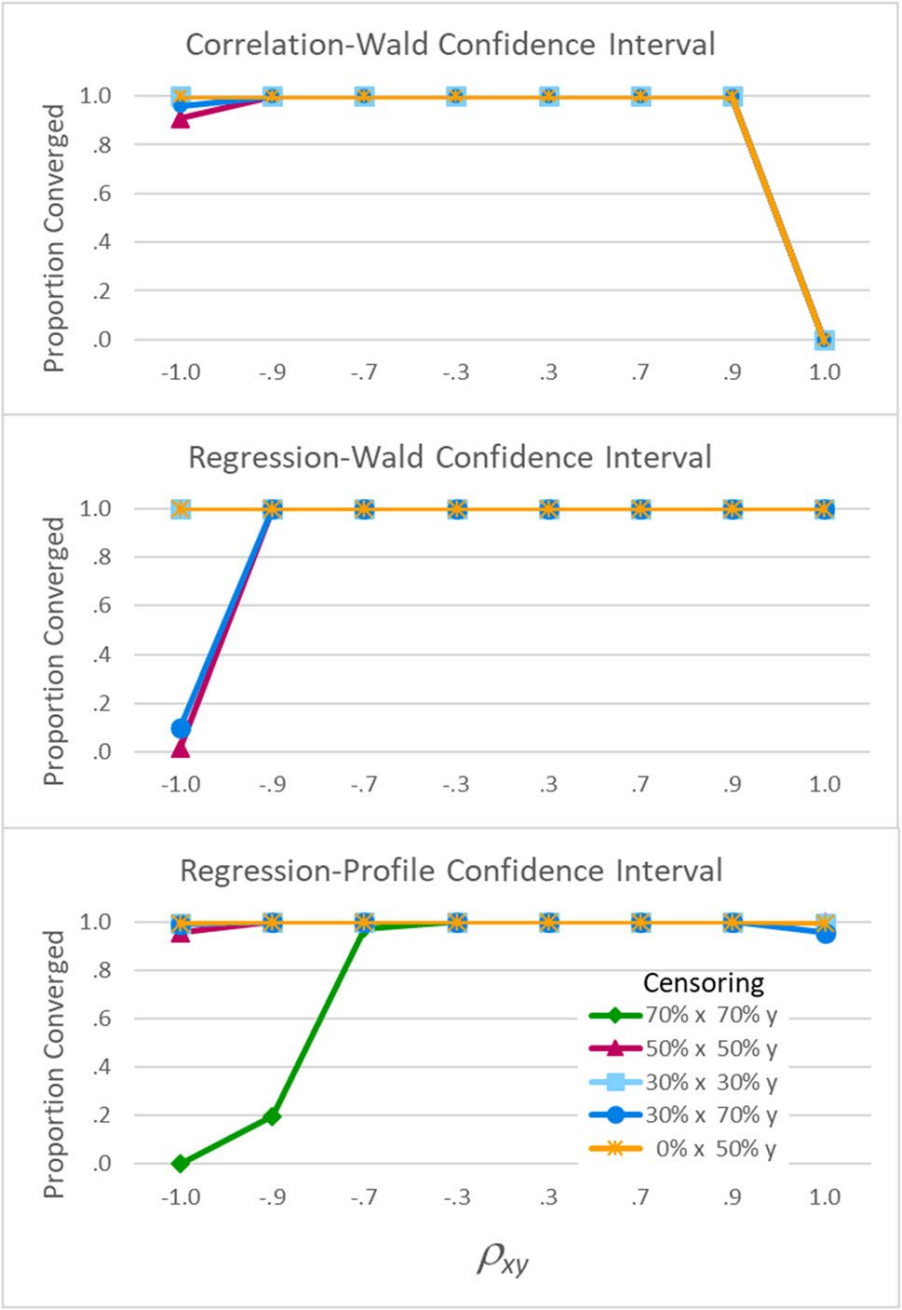
Proportion of trials that converged upon confidence interval estimates. All three confidence intervals sometimes failed to converge when *ρ*_*XY*_ = ±1 (on the far left and far right of each panel). The regression-profile confidence intervals (bottom panel) also sometimes failed to converge when *ρ*_*XY*_ ≠ ±1

None of these confidence interval methods converged upon solutions for all trials of all cells. Figure 7 shows that all three methods sometimes failed to converge when *ρ*_*XY*_ =  ± 1 (on the far left and far right of each panel).

Of greater concern is the fact that the regression-profile confidence intervals (bottom panel) also sometimes failed to converge for values that might actually occur in research settings. Specifically, when there was 70% censoring on both variables (the green diamond-marked line), regression-profile confidence intervals failed to converge 2.8% of the time for *ρ*_*XY*_ =  − .7 and 80.4% of the time when *ρ*_*XY*_ =  − .9. Such failures would limit the usefulness of regression-profile confidence intervals with empirical data.

In the rest of this paper, we include summary statistics for all available cells in our figures, tables, and text. However, data are missing for those cells where *lava* failed to converge for any of the 2000 trials. This occurred for several cells where *ρ*_*XY*_ =  ± 1. Moreover, we could not include those cells in our between-within ANOVAs. However, omitting *only* these cells would cause a lack of cell proportionality, which would skew significance tests for main effects and interactions. Therefore, we excluded all cells where *ρ*_*XY*_ =  ± 1 from subsequent analyses.

##### Proportion of confidence intervals that included ρ_XY_

**Fig. 8:**
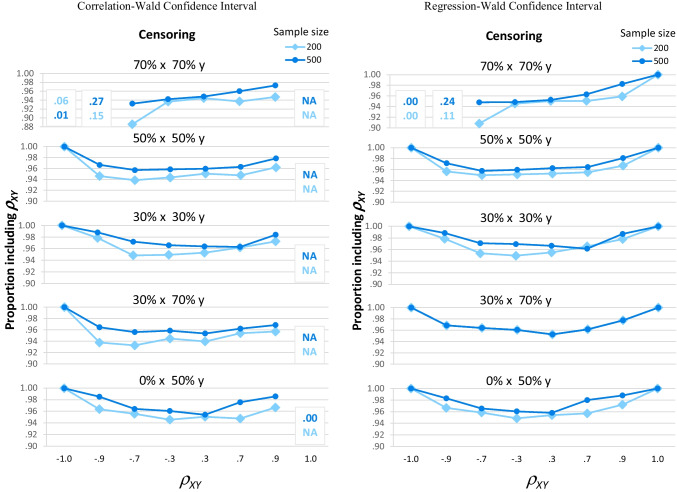

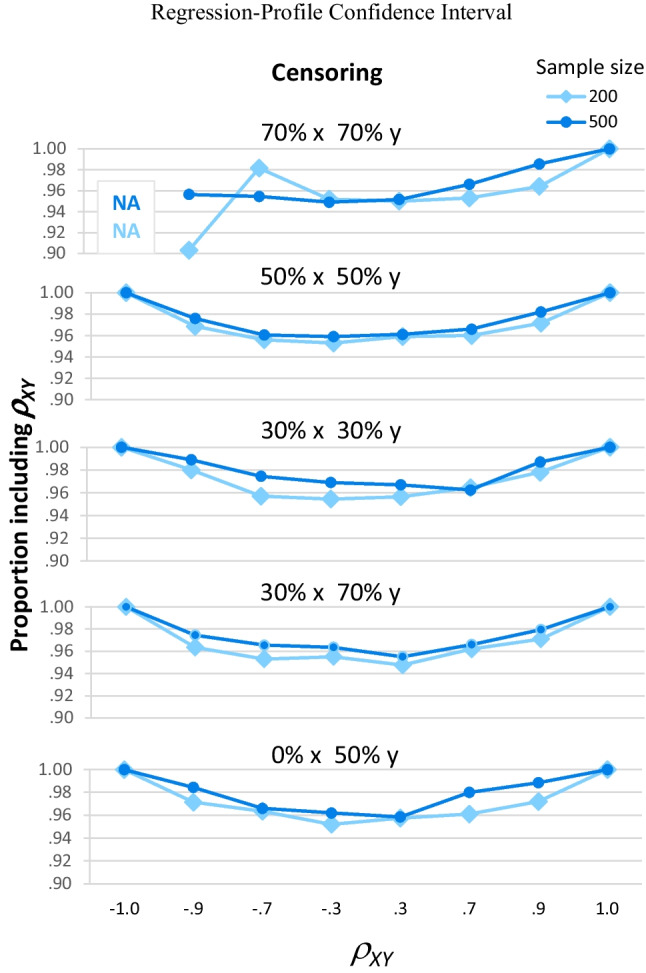
Proportion of confidence intervals including *ρ*_*XY*_. All three types of confidence intervals included *ρ*_*XY*_ at least 94% of the time, except for two types of data. First, when *ρ*_*XY*_ = 1 (far right), the correlation-Wald confidence intervals (first panel) never included *ρ*_*XY*_. Second, when there was 70% censoring on both variables (top section of each panel) and large negative correlations (left-hand side), the confidence intervals rarely included *ρ*_*XY*_

All three confidence interval methods worked well for the types of data most likely to be encountered in practice. When *ρ*_*XY*_ was between −.9 and .9 and there was no more than 50% censoring on either variable, Fig. 8 shows all three confidence intervals methods included *ρ*_*XY*_ at least 94% of the time regardless of sample size.

Differences between the confidence interval methods appear when we consider extreme data. These differences are reflected in a significant interaction of confidence interval method, *ρ*_*XY*_, sample size, and censoring pattern, *F*(40,118) = 4.76, *p* < .001, partial eta-squared = .617.

When *ρ*_*XY*_ = 1, the regression-Wald intervals were better than both of the alternatives. The correlation-Wald intervals (Fig. 8 first panel, right-hand side) converged for only one of the 20,000 trials (Fig. 7 first panel, right-hand side). In that one trial, *ρ*_*XY*_ = 1, but the interval was estimated as [−1.03, −1.03] and thus provided no information about the true value of *ρ*_*XY*_ (being both outside the allowable value for a correlation and in the wrong direction). The regression-profile intervals (third panel) did better: They *usually* converged (Fig. 7), and when they converged, always included *ρ*_*XY*_ (Fig. 8). The regression-Wald intervals (second panel) performed even better for this type of data: They *always* converged (Fig. 7) and always included *ρ*_*XY*_ (Fig. 8).

When *ρ*_*XY*_ =  − 1 and there was 70% censoring on both variables, the regression-profile intervals never converged (Fig. 7 third panel, left-hand side), and so these cells are not shown in Fig. 8. In contrast, the Wald intervals converged 100% of the time (Fig. 7 first and second panels) and were consistently close to *ρ*_*XY*_. They were centered at −.98 with a mean width of .01 or .02. Thus, although they included *ρ*_*XY*_ only rarely (4% of the time for correlation-Wald in the first panel of Fig. 8; 0% for regression-Wald in the second panel), the Wald intervals were far superior to the regression-profile intervals for this type of data.

When *ρ*_*XY*_ =  − .9 and there was 70% censoring on both variables, the correlation-Wald (Fig. 8 first panel) and regression-Wald (second panel) intervals captured *ρ*_*XY*_ less often than the regression-profile intervals. Specifically, the two Wald interval methods included *ρ*_*XY*_ only 11% to 15% of the time for *n* = 200 (light diamond-marked lines) and 24% to 27% of the time for *n* = 500 (dark circle-marked lines), whereas the regression-profile intervals included *ρ*_*XY*_ 90% of the time when *n* = 200 and 95% of the time when *n* = 500.

Typically, high coverage rates are desirable. However, when *ρ*_*XY*_ =  − .9 and there was 70% censoring on both variables, these confidence intervals also differed in their locations and widths. In terms of locations, Supplemental Table 1 shows the Wald intervals tended to be centered around −.95, making the relations look somewhat stronger than they actually were. In contrast, the regression-profile intervals were centered around –.78, making the relations look much weaker than they actually were. In terms of widths, the next section will reveal important differences between the three methods for this type of data.

##### Average width of the confidence intervals

**Fig. 9:**
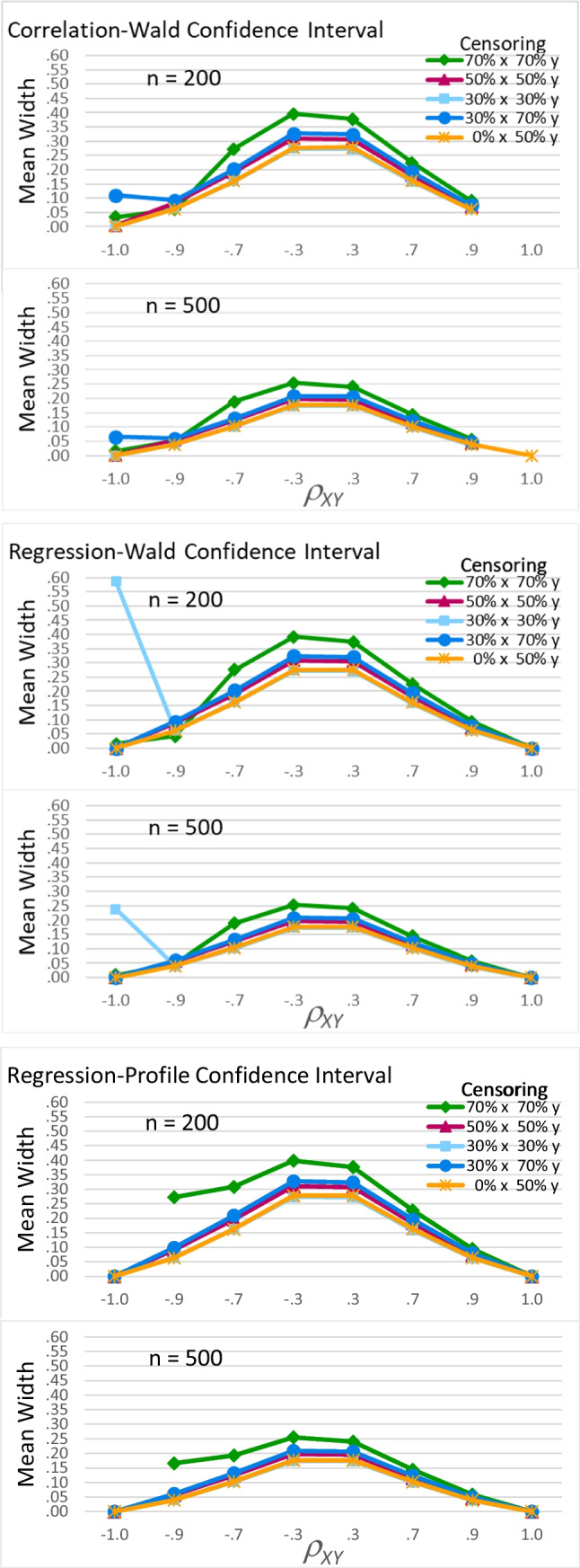
Mean confidence interval width. This figure shows the mean width of all calculated confidence intervals; if a data point for a particular combination of sample size, *ρ*_*XY*_, and censoring pattern is absent, this indicates *lava* never converged upon an estimate for that combination of factors

Once again, the three confidence interval methods had similar performance for the types of data most likely to be encountered in practice: When *ρ*_*XY*_ was between −.9 and .9 and there was no more than moderate censoring, mean widths from the three methods followed the same pattern. Figure 9 shows the three types of confidence intervals all had larger mean widths when *ρ*_*XY*_ was smaller (near the center of each graph; *F*(5, 60) = 379111.52, *p* < .001, partial eta-squared = 1), when there was more censoring (the darker lines in each graph; *F*(4, 60) = 51853.61, *p* < .001, partial eta-squared = 1), and when sample size was smaller (the top half of each panel; *F*(1, 60) = 384906.80, *p* < .001, partial eta-squared = 1). For example, when *n* = 200, for large *ρ*_*XY*_ (±.9), widths varied from .05 to .10 for most cells; for small *ρ*_*XY*_ (±.3), widths varied from .27 to .33 for most cells. When *n* = 500, for large *ρ*_*XY*_ (±.9), widths varied from .04 to .06 for most cells; for small *ρ*_*XY*_ (±.3), widths varied from .17 to .22 for most cells. In addition, these three factors interacted, *F*(20, 60) = 71.84, *p* < .001, partial eta-squared = .960: The interaction of censoring pattern and sample size was larger for small *ρ*_*XY*_.

Differences between the confidence interval methods are again noticeable when we consider extreme data, which again led to a significant interaction of confidence interval method, *ρ*_*XY*_, sample size, and censoring pattern, *F*(40,118) = 88.79, *p* < .001, partial eta-squared = .986. When *ρ*_*XY*_ =  − .9 and there was 70% censoring on both variables (green diamond-marked lines), the Wald intervals (top two panels) were narrow, with mean widths near .05. In contrast, the regression-profile intervals (bottom panel) were three to five times as wide, with widths of .27 when *n* = 200 and .17 when *n* = 500. Thus, the higher coverage rates seen for the regression-profile intervals for this type of data (Fig. 8) were only achieved by making these intervals much wider (Fig. 9), which decreases the intervals’ precision and usefulness.

When *ρ*_*XY*_ = 1, the regression-Wald intervals were better than both of the alternatives. The correlation-Wald intervals (top panels) never converged upon meaningful solutions (Fig. 7) and so are usually not shown in Fig. 9. The regression-profile intervals (bottom panel) performed much better: They converged most of the time (about 95% of the time when there was 30% censoring on *x* and 70% on *y*, and at least 99% of the time for other censoring patterns), and when they converged, the intervals were always estimated with perfect accuracy and precision as being [1,1]. The regression-Wald intervals (middle panels) were the best: They always converged and were always estimated as [1,1].

When *ρ*_*XY*_ =  − 1, all three confidence interval methods performed poorly for some patterns of censoring, but they varied in the patterns of censoring that caused problems and in the severity of those problems. Most disastrously, the regression-profile intervals did not converge upon solutions when there was 70% censoring on both variables (and so no widths are shown for those cells in the bottom panel of Fig. 9). Nearly as bad, the regression-Wald intervals had substantially larger mean widths (compared to cells with other patterns of censoring) when there was 30% censoring on both variables (middle panel of Fig. 9, light blue square-marked line). Less problematic, the correlation-Wald intervals had noticeably larger mean widths (compared to cells with less censoring) when there was 30% censoring on *x* and 70% on *y* (top panel of Fig. 9, dark blue circle-marked line). For both Wald methods, these larger mean widths occurred where some intervals had widths greater than or equal to 2 (see Supplemental Table 1).

### Discussion

Data point censoring may affect correlations in a variety of psychological studies. Negative correlations can be pulled substantially closer to 0 when two variables both have left censoring or both have right censoring. Conversely, positive correlations can be pulled substantially closer to 0 when one variable has left censoring and the other right. If these correlations are not corrected, relations between variables will be mischaracterized, with downstream consequences such as incorrect structural models and inappropriate decisions regarding the number of dimensions.

CensorCorr (Barchard, 2022) can be used to model the effects of data point censoring on correlations. As a teaching tool, CensorCorr can show students the difference between right and left censoring and their differential effects on positive and negative correlations. As a research tool, CensorCorr can be used to find theoretical correlations between uncensored variables (*ρ*_*XY*_) that are compatible with both the observed correlations between censored variables (*r*_*xy*_) and observed floor and ceiling effects that may have been caused by censoring.

To correct for the distorting effects of censoring, Holst and Budtz-Jørgensen (2013) developed maximum likelihood (ML) estimators for the covariances among uncensored variables based upon an extension of tobit regression and the assumption that uncensored variables have a multivariate normal distribution. They implemented the ML estimators in the R package *lava*, thus making it easy for others to use. In 2015, Holst and colleagues conducted a simulation study to show that the ML estimators, as implemented in *lava*, work well for one structural equation model. Though these results are encouraging, no systematic research has examined the performance of the ML estimators or their implementation in *lava*, not even for the simplest case of estimating a correlation when the assumption of multivariate normality had been met. And, of course, no research has compared the several possible ways of estimating a correlation in *lava*. Therefore, the purpose of this study was to examine the accuracy of the ML estimators for correlations among uncensored variables and to compare the different ways these estimators have been implemented in *lava*.

Our simulation study examined the accuracy and precision of two point estimates and three interval estimates of *ρ*_*XY*_, using different values of *ρ*_*XY*_ and sample size, and various patterns of left censoring. We found that the ML estimates of *ρ*_*XY*_ worked remarkably well for most types of data examined, but that there were important differences depending upon how the estimates were implemented in *lava*.

#### Point estimates

The point estimates of *ρ*_*XY*_ from the correlation and regression models had similar performance. Unless there was severe censoring, both types of estimates were accurate and precise. These estimates had little bias, typically smaller than ±.005. Estimates also had relatively small values of RMSE, which decreased further as sample size increased from 200 to 500. Indeed, RMSE based upon censored data was similar to RMSE for the regular Pearson product-moment correlation based upon uncensored data (Table 2). Therefore, for most normally distributed variables, sample sizes of 200 may be sufficient to provide precise and unbiased estimates of *ρ*_*XY*_ from censored data on *x* and *y*.

**Table 2:** RMSE of estimates of ρ_XY_ for various degrees of censoring

Sample Size	Average Censoring	*ρ*_*XY*_							
		−1	−.9	−.7	−.3	.3	.7	.9	1
200	0%	.00	.03	.05	.07	.07	.05	.03	.00
	≤ 30%	.01	.02	.04	.07	.07	.04	.02	.00
	50%	.00	.02	.05	.08	.08	.05	.02	.00
	70%	.02	.08☨	.10☨	.11	.10	.06	.02	.00
500	0%	.00	.02	.03	.04	.04	.03	.02	.00
	≤ 30%	.01	.01	.03	.04	.04	.03	.01	.01
	50%	.00	.02	.03	.05	.05	.03	.01	.00
	70%	.02	.07☨	.05	.07	.06	.04	.01	.00

*Note.* When there was no censoring (first row for each sample size), RMSE for *r*_*XY*_ was calculated as $\sqrt{{\left( standard\ error\right)}^2+{(bias)}^2}$, where the standard error was calculated as $\sqrt{\left(1-{\rho}^2\right)/n-2}$ and bias was approximated as $-{\rho \left(1-{\rho}^2\right)}/{2n}$ (Hotelling, 1953). When there was censoring (remaining rows), RMSE was calculated using the results of the simulation study reported here by averaging values across the correlation and regression models. ☨ In dagger-marked cells, RMSE for censored data was notably larger than RMSE for uncensored data

However, when there was 70% censoring on both *x* and *y*, both point estimates performed poorly. RMSE was larger, particularly for negative correlations, and did not decrease much for *ρ*_*XY*_ =  − .9 when sample size increased to 500. In addition, these estimates were positively biased for *ρ*_*XY*_ =  − 1 and negatively biased for *ρ*_*XY*_ =  − .9. These two biases would make it difficult to distinguish true correlations that fall between −.9 and −1. Therefore, if researchers are interested in strong negative correlations between variables that may have left censoring, they should ideally design their measurements to have 50% censoring or less on both variables. For example, if researchers want to test if two variables are opposites (and have a correlation that is near −1 times the square root of the product of their reliabilities), they should endeavor to obtain precise (uncensored) scores for even small amounts of their variables.

Despite the similarities between the two models in terms of bias and precision, we recommend the regression model. The correlation model provided reasonable estimates of *ρ*_*XY*_ for most data likely to be encountered in practice. However, it might not work so well when its assumptions have been violated (for example, when data are not normally distributed or are discrete). Moreover, even when its assumptions were met, the correlation model sometimes failed to converge upon a solution and sometimes provided estimates that were outside the allowable range for a correlation. To prevent invalid estimates, Holst (personal communication, April 2020) recommended using the regression model because it allows researchers to constrain the solution to guarantee the final estimate is within the allowable range. The present study demonstrated that these constraints successfully prevented invalid estimates of *ρ*_*XY*_ and did not distort estimates in any way. Therefore, there appears to be no disadvantage to using the model that is guaranteed to provide valid estimates: We recommend researchers obtain point estimates of *ρ*_*XY*_ using the constrained regression model.

#### Interval estimates

All three confidence intervals worked well for most types of data. All three worked well when *ρ*_*XY*_ was between −.3 and −.9 as long as there was no more than 50% censoring on either variable and also when *ρ*_*XY*_ was between .3 and .9. They converged upon solutions 100% of the time, included *ρ*_*XY*_ at least 94% of the time, and were relatively narrow. This is remarkable performance given the strong effect censoring can have on correlations.

When there was only moderate censoring (e.g., 30% censoring on both variables or 50% censoring on just one variable), these confidence intervals had widths that were comparable to the widths of confidence intervals for simple bivariate correlations when there is no censoring (Table 3). Thus, when there is only small to moderate censoring, sample sizes of 200 may be sufficient to obtain relatively precise interval estimates of *ρ*_*XY*_. However, when there was a high degree of censoring (e.g., an average of at least 50% censoring across the two variables), the confidence intervals based upon censored data were substantially wider than those based upon uncensored data. Therefore, researchers need to either minimize censoring or use larger sample sizes to provide precise interval estimates.

**Table 3:** Width of confidence intervals for ρ_XY_ for various degrees of censoring

Sample size	Average censoring	*ρ*_*XY*_			
		±1	±.9	±.7	±.3
200	0%	.000	.053	.142	.252
	≤ 30%	.049	.063	.161	.275
	50%	.009	.083	.194	.317
	70%	.012	.109	.256	.386
500	0%	.000	.033	.089	.158
	≤ 30%	.020	.039	.102	.175
	50%	.006	.053	.123	.202
	70%	.006	.073	.167	.247

*Note.* When there was no censoring (first row for each sample size), the width of the confidence interval for *ρ*_*XY*_ was calculated using Lowry (n.d.), which uses the Fisher *r*-to-*z* transformation. When there was censoring (remaining rows), the width of the confidence interval for *ρ*_*XY*_ was calculated using the results of the simulation study reported here by averaging values across the correlation and regression models.

Two types of data caused particular problems for the confidence intervals. First, when *ρ*_*XY*_ =  ± 1, the intervals often failed to converge upon a solution. However, for each combination of *ρ*_*XY*_ and censoring pattern, there was always one method that provided consistently accurate intervals. Researchers can use these results to select which confidence interval to calculate if ever they obtain a point estimate ${\hat{\rho}}_{XY}$ that equals ±1. If ${\hat{\rho}}_{XY}=1$, use the regression-Wald interval. If ${\hat{\rho}}_{XY}=-1$, use the regression-Wald interval if there is at least 50% censoring (on average) across the two variables, otherwise use the regression-profile interval.

Second, the confidence intervals had problems when *ρ*_*XY*_ =  − .9 and there was 70% censoring on both variables. This kind of data caused problems for all three confidence interval methods. The regression-profile intervals performed badly. Although these intervals captured *ρ*_*XY*_ 90–95% of the time (the highest proportions of any confidence intervals), they were very wide (mean width of .27 for *n* = 200 and .17 for *n* = 500), providing little information about the location of *ρ*_*XY*_. Moreover, the regression-profile intervals only converged upon a solution 13% of the time for *n* = 200 and 26% of the time for *n* = 500. Thus, the regression-profile method did not produce good confidence intervals for large negative correlations under severe censoring.

The correlation-Wald confidence intervals performed better than the regression-profile intervals for this type of data. Although the correlation-Wald intervals were biased (centered around −.95) and rarely included *ρ*_*XY*_ (only 15% of the time for *n* = 200 and only 27% of the time for *n* = 500), these intervals were narrow (mean width .06), successfully conveyed that the correlation was large and negative, and – unlike the regression-profile intervals – always converged upon a solution.

Finally, the regression-Wald intervals performed even better than the correlation-Wald intervals for this kind of data. The regression-Wald intervals were similar to the correlation-Wald in that they always converged upon a solution and successfully conveyed that the correlation was large and negative. The regression-Wald intervals also had a similar level of bias (−.05) and were slightly narrower on average (.04 vs. .06). However, the methods differed in terms of their maximum widths: The regression-Wald intervals were roughly one third as wide as the correlation-Wald (e.g., when *n* = 200, widths of .37 vs. 1.06; when *n* = 500, .22 vs. .41), and thus were sometimes much more precise and informative.

Comparing results across the three confidence interval methods, we conclude the regression-Wald intervals were the best when variables have extreme left censoring and large negative correlations. Given that the three methods were otherwise comparable for the types of data likely to be encountered in empirical research, we recommend the regression-Wald confidence intervals be used for all data types (unless ${\hat{\rho}}_{XY}=\pm 1$).

#### Implications for other censoring patterns

Although the current simulations used two variables that were both left censored, right-censored data can be analyzed using functions designed for left-censored data: The data are simply inverted first. We can therefore deduce the accuracy and precision of point and interval estimates of *ρ*_*XY*_ for other combinations of right and left censoring. If both variables are right censored, identical results will occur: Censoring will have its largest effect on negative correlations, and *lava* estimates will be unbiased and precise unless (a) *ρ*_*XY*_ =  ± 1 or (b) *ρ*_*XY*_ is strong and negative and there is severe censoring. If instead one variable is right censored and the other is left censored, opposite results will occur: Censoring will have its largest effect on *positive* correlations, and *lava* estimates will be unbiased and precise unless (a) *ρ*_*XY*_ =  ± 1 or (b) *ρ*_*XY*_ is strong and *positive* and there is severe censoring.

#### Conclusions

When researchers suspect their data may have been censored, we recommend they estimate correlations using the ML estimates developed by Holst and Budtz-Jørgensen (2013), which have been implemented in *lava*. We recommend researchers use the regression model to provide point estimates of *ρ*_*XY*_ and the regression-Wald method to provide interval estimates. However, researchers should keep in mind that these estimates are biased for large negative values of *ρ*_*XY*_ when there is severe left censoring on both variables (or severe right censoring on both) and that they are similarly biased for large positive values of *ρ*_*XY*_ when there is severe left censoring on one variable and severe right censoring on the other. Therefore, if researchers are interested in large correlations, we recommend they try to minimize censoring. For example, they can avoid floor and ceiling effects by using measures that are appropriate to the population being examined and minimize the effects of response biases by using anonymous or confidential responses. Additionally, they can reduce the level of detection by using fine-grained measurement procedures (e.g., partial credit, 100-point rating scales, or more powerful microscopes). In longitudinal studies, they can observe a larger portion of the target events (including low frequency events) by running their studies longer. Minimizing censoring in these and other ways will reduce bias when estimating large correlations in the presence of censoring.

Additional research is needed. Only one other study (Holst et al., 2015) has examined the performance of the *lava* package and its ML estimates: That study examined a single structural model and, as was the case in the current study, all variables had normal distributions or underlying normal distributions. While it is essential to first check if a procedure works under the circumstances for which it was designed (e.g., normality), we encourage future research using additional structural models (including confirmatory factor analysis and longitudinal analysis) and a variety of data distributions (including skewed and polychotomous data).

Additionally, future research could examine other implementations of the ML estimates developed by Holst and Budtz-Jørgensen (2013). This study examined the performance of the ML estimates as implemented in the R package *lava* (Holst, 2020a), using the code described in the appendix and given in the supplementary materials. However, the differences between the two point estimates and between the three interval estimates highlight the importance of how statistical methods are programmed. Therefore, other implementations of the ML estimates should be explored. For example, the current simulations used the default optimizer (*nlminb*), but other optimizers could be used. The current simulations constrained ${\hat{\rho}}_{XY}$ in the regression model, but different constraints could be used. And the current simulations examined left censoring (and by extension right censoring), but *lava* now models interval censoring, which has yet to be examined. Finally, alternative programs could be created to implement the ML estimators, either starting from scratch or using extensions of the *lava* and *mets* packages (GitHub, n.d.). In the meantime, given the strong performance of the ML estimates in both this study and the study by Holst et al. (2015) – including, for most datasets, estimates of *ρ*_*XY*_ with bias of less than ±.005 and RMSE values similar to those obtained when there is no censoring – we recommend *lava* be used to improve the accuracy and precision of estimates of the relations among censored variables.

## Data Availability

All data generated or analyzed during this study are included in this published article and its supplementary information files, which are available at https://osf.io/exktg/.

## Appendix: How to use *lava* to estimate the correlation between uncensored variables

*Load packages and set tolerance*

The *lava* package requires the *mets* package (Holst, 2020b), so we must load both.

*Lava* uses the Moore-Penrose inverse, so we must set its tolerance.

*Prepare our data*

*Lava* needs to know which data points were censored. Specifying which were censored requires three steps. First, to simulate left-censored data, we need to specify the lower limits for *x* and *y*. Our simulation study varied the lower limits based upon the degree of censoring. However, in this example, we set the lower limits for both *x* and *y* as 1.

Second, we need to create indicator variables to specify whether each value of *x* and *y* was observed. We call these variables *obsX* and *obsY*. We set them to FALSE when the values of *x* and *y* are equal to the *x* and *y* lower limits. Otherwise, we set them to TRUE.

Third, we need to create a single variable indicating the value of *x* when it was observed, the value of *x* when it was censored, and whether it was left or right censored. Similarly, we need to create a single variable for the values and censoring information for *y*. To do this, we use the *Surv* function from the *mets* package. Then we assemble these variables into a data frame.

We will hand this data frame to *lava* to tell it which data points were censored.

*Specify and estimate the models*

Two models can be used to estimate the correlation between *X* and *Y*. The first is the correlation model, which is straight-forward to specify and estimate.

Using the estimates from the correlation model, we calculate a Wald confidence interval for *ρ*_*XY*_.

The second is the constrained regression model. To set up this model, first we specify a regression model. Second, we constrain that model to prevent estimates from falling outside the allowable range for a correlation (−1 to +1). Third, we require the Fischer’s *r*-to-*z* transformation. Finally, we back-transform the results (from *z* to *r*) to estimate the correlation.

The regression model allows two types of confidence intervals, the Wald confidence interval and the profile confidence interval. The Wald confidence interval is calculated by back-transforming the end points of the confidence interval for *z*. Because this transformation (the hyperbolic tangent) is nonlinear, the confidence interval for the correlation may be slightly asymmetric, especially for wide intervals.

The profile confidence intervals can be difficult to calculate when *ρ*_*XY*_ is near ±1, because Fisher’s *z*-score transformation of *r* (the inverse hyperbolic tangent) approaches ±infinity as *r* approaches ±1. To facilitate successful calculation of the profile confidence intervals, Holst (personal communication, April 2020) suggested *z*-scores be kept within the boundaries ±10, leading to *r*_*XY*_ boundaries of ±.999999996 (which equal ±1 when rounded to eight decimal places). However, preliminary analyses showed *lava* sometimes failed to converge upon a solution when using those boundaries. Boundaries of ±3 resulted in convergence more frequently, without reducing the proportion of the confidence intervals that included *ρ*_*XY*_. Stricter limits did not further improve convergence. Therefore, we examined profile confidence intervals with *z*-score boundaries of ±3, leading to *r*_*XY*_ boundaries of ±.99505 (which equal ±1 when rounded to two decimal places – the usual number of decimals reported in psychological research).

## Supplemental Materials

ESM 1 (DOCX 20 kb)

## Footnotes

1 Formula 3 and 4 correct a small mathematical error in Formula 17 from Carroll (2000).

2 To see these relationships, substitute the names of the variables under consideration for the names given in Fig. 3. For example, to see that *ρ*_*ac*_ is estimated as .651 when *ρ*_*AC*_ = .707, substitute *a* and *A* for *x* and *X*, and substitute *c* and *C* for *y* and *Y*; look at the horizontal axis to see where *ρ*_*XY*_ (now *ρ*_*AC*_) is .707, and then read off the value of *r*_*xy*_ (now *r*_*ac*_).

3 At the time of this writing, no other multivariate program uses methods designed specifically for censored data. For example, the *OpenMx* manual (Boker et al., 2022) does not mention censored data and the *lavaan* manual (Rosseel et al., 2022) states it does not support censored variables. Mplus is frequently used to analyze censored data, but it uses either maximum likelihood methods that are robust to violations of normality (MLM) or weighted least square estimates (WLSMV) that treat censored data as ordinal variables (Muthén, 2001; Muthén & Muthén, 2017), rather than methods designed specifically for censored data.

4 This method was designed for ordered categorical variables (Muthén, 1984), but is frequently used to estimate relations between censored variables Muthén & Muthén (1998-2017).

5 *Randomisation validity* occurs when the coverage proportion is near nominal; *confidence validity*, when it is at least nominal (Morris et al., 2019). We examine confidence validity when widths are consistently 0: In those cells, either all confidence intervals include *ρ*_*XY*_ or none do.

6 To determine if the correlation- and regression-model estimates of *ρ*_*XY*_ are consistent (i.e., approach the population parameter *ρ*_*XY*_ as sample size increases), we ran 10 trials with sample sizes of 100,000. For both models, when *ρ*_*XY*_ =  − .9 and there was 70% censoring on both variables, ${\hat{\rho}}_{XY}$ was now unbiased. However, when *ρ*_*XY*_ =  − 1, ${\hat{\rho}}_{XY}$ was still biased: Mean ${\hat{\rho}}_{XY}$ still equaled −.983 for both models. See the bottom two rows of Supplemental Table 1.

## Code availability

The original R script used to run the Monte Carlo study is not available; however, a neatened version of this script is available at https://osf.io/exktg/ and can replicate the reported study results to within the accuracy of the random number generator. All code used to analyze the data for this study are available at https://osf.io/exktg/. The code for the R package *lava* is available at https://cran.r-project.org/package=lava.
