# Supplementary material for: Distorted correlations among censored data: causes, effects, and correction
Source: Behav Res Methods. 2023 Dec 21;56(3):1207–28. doi: 10.3758/s13428-023-02086-5 (PMC10991075; doi:10.3758/s13428-023-02086-5)
Supplement: ESM 1 — (DOCX 20 kb) [file 13428_2023_2086_MOESM1_ESM.docx]

Supplemental Table 1 and computer readable results from all cells: <https://osf.io/exktg/>

R script and csv file to replicate the Monte Carlo study: <https://osf.io/exktg/>

SPSS syntax and output files: <https://osf.io/exktg/>

CensorCorr: <https://osf.io/pfqy2/>
